# Soil chemical and functional indicators reveal limitations of restoration measures in abandoned metal mine soils of SE Spain: implications for ongoing and future management

**DOI:** 10.1007/s10653-026-03378-3

**Published:** 2026-07-22

**Authors:** Matías Ceacero-Moreno, José Álvarez-Rogel, M. Nazaret González-Alcaraz

**Affiliations:** Department of Agricultural Engineering of the E.T.S.I.A., Technical University of Cartagena Member of European University of Technology EUT+, 30203 Cartagena, Spain

**Keywords:** Soil pollution, Mine tailings, Metalliferous wastes, Rare earth elements, Soil functionality, Mining landscape

## Abstract

Abandoned metal mine tailings remain long-term sources of contamination, and restoration measures aim to reduce contaminant mobility while supporting the recovery of key soil functions. This study evaluated the chemical and functional status of mine wastes, capping materials, and surrounding soils associated with previously restored mine tailings in the La Unión-Sierra de Cartagena mining district (SE Spain). Soils exhibited highly contrasting chemical conditions, ranging from strongly acidic to slightly alkaline (pH 2.6–7.8), with electrical conductivity reaching 10.5 dS m^−1^ in mining-affected sites. Total concentrations of Mn, Zn, Pb, Cd and As were high, reaching up to 35,960, 11,760, 36,340, 26 and 274 mg kg^−1^, respectively. Maximum concentrations exceeded regional Generic Values of Reference by up to two orders of magnitude for Mn and As, and by more than three orders of magnitude for Pb. However, total concentrations were poor predictors of contaminant mobility. Water- and ammonium acetate-extractable fractions revealed substantial metal(loid) availability in some capped soils, indicating that previously implemented restoration measures did not fully prevent contaminant mobilization. Rare earth elements were not enriched by mining activity, although enhanced mobility was observed locally under extremely acidic and saline conditions. Some capped soils exhibited signs of functional recovery. Carbonate-rich capping combined with vegetation establishment promoted partial recovery of organic matter pools and microbial activity, whereas mine wastes and sites capped with coarse mine-derived materials remained severely impaired. Overall, the results highlight the importance of integrating chemical availability and functional indicators to assess restoration effectiveness and guide management strategies in semi-arid mining environments.

**Supplementary Information:**

The online version contains supplementary material available at 10.1007/s10653-026-03378-3.

## Introduction

Metal mining generates vast amounts of hazardous waste, often stored in tailings that are abandoned and act as long-term sources of environmental contamination (FAO & ITPS, 2015). Potential restoration strategies include engineering-based interventions (e.g., inertization, encapsulation, leaching, or waste capping; Tordoff et al., 2000) as well as nature-based solutions such as phytostabilization or assisted revegetation (Mendez & Maier, 2008). The selection of an appropriate restoration strategy should be guided by factors such as contaminant type and load, the spatial extent of the impact, topography, proximity to water bodies or human settlements, the ecological value of the site, and the availability of financial and technical resources (Bradshaw, 1997). In all cases, restoration measures should ensure the long-term physical and chemical stability of mine wastes to minimize persistent risks to environmental quality and human health (Edraki et al., 2014). Nevertheless, non-restored abandoned mine tailings remain widespread and may continue to cause diffuse contamination long after mine closure through the mobilization of contaminated water by runoff and the dispersion of contaminated sediments by wind and water erosion (Conesa & Schulin, 2010; Dudka & Adriano, 1997).

This situation is well illustrated by the former mining district of La Unión-Sierra de Cartagena (Southeast Spain; Fig. S1, Suppl. Mat.), which has a long history of metal mining dating back to Roman times. The district hosts polymetallic sulfide deposits associated with the metamorphic units of the Internal Betic Zone and has been historically exploited mainly for Pb–Zn mineralization dominated by galena (PbS) and sphalerite (ZnS), together with other sulfide minerals such as pyrite (FeS_2_) and marcasite (FeS_2_) (Manteca & Ovejero, 1992). Mining activity intensified markedly during the early twentieth century, generating large volumes of ore-processing waste enriched in potentially toxic elements such as Cd, Pb, Zn and As. Ore processing often involved crushing, grinding and flotation techniques to concentrate the valuable sulfide minerals, generating large quantities of fine-grained wastes that were commonly deposited in open-air tailings disposal facilities. Although mining activities ceased in 1991, dozens of abandoned tailings remain across the area and continue to represent a source of contamination for surrounding soils, surface waters, and the nearby Mar Menor lagoon (the largest coastal lagoon in the Mediterranean basin) (Conesa & Schulin, 2010; Martínez-López et al., 2021). Previous studies have reported limited effectiveness of some restoration measures implemented in the area, particularly when interventions were superficial or poorly adapted to the characteristics of the mine waste (Gómez-Ros et al., 2013).

In response to the environmental risks posed by abandoned mine waste in the La Unión-Sierra de Cartagena mining district and its potential contribution to the degradation of the Mar Menor lagoon, the Spanish Government, through the Ministry for the Ecological Transition and the Demographic Challenge (MITECO), has recently launched large-scale restoration projects at several mining sites within the Mar Menor Priority Actions Framework (NextGenerationEU) (MITECO, 2023, 2024). The sampling campaign was conducted before the implementation of these large-scale restoration works, which include reconfiguration and stabilization of mine waste deposits, installation of sealing (capping) systems and subsequent revegetation.

Assessing restoration success in mine soils remains challenging because no single criterion can fully capture the complexity of recovery processes. Recent studies in arid and semi-arid mining environments have emphasized the need for integrated assessment approaches that consider not only contaminant stabilization but also the recovery of soil quality and ecosystem functioning (Mou et al., 2025; Zhu et al., 2024). This broader perspective extends beyond the traditional emphasis on reducing contaminant concentrations and mobility in restored mining areas (Bolan et al., 2014; Mench et al., 2010). Indicators related to organic matter dynamics, microbial activity and nutrient cycling can provide valuable information on ecosystem recovery beyond that obtained from purely chemical criteria, as recently demonstrated in restored and spontaneously colonized metal mine tailings from semi-arid environments (Ceacero-Moreno et al., 2025).

Within this framework, the present study assessed whether previously implemented restoration actions in selected areas of the La Unión-Sierra de Cartagena mining district had resulted in acceptable soil chemical quality and functionality. The evaluation focused on representative sites in which restoration measures had been implemented, mainly involving the capping of mine tailings with externally sourced materials and, at some sites, the establishment of vegetation cover. Soil physicochemical and biological properties, together with total and available concentrations of metal(loid)s, were analyzed to assess the effectiveness and limitations of past restoration measures. Special attention was given to functional soil indicators related to organic matter dynamics and nutrient cycling, including carbon and nitrogen mineralization, as proxies of microbial activity and ecosystem recovery. Furthermore, given the limited information available on rare earth elements (REEs) in the study area (e.g., Rodríguez-Pacheco et al., 2023), a range of REEs was also included to provide additional information on their concentrations in restored and non-restored mine soils and to contribute to the currently limited knowledge available for the La Unión-Sierra de Cartagena mining district.

## Material and methods

### Study sites and sampling

Four zones with contrasting mining legacies were selected for this study (Fig. S1, Suppl. Mat.). Within each zone, several sampling sites were selected to represent different substrate and restoration conditions. At each site, five composite surface soil samples (0–20 cm depth; five subsamples each) were collected at distances of approximately 10 m. Throughout the manuscript, the term soil is used to refer to all sampled surface materials, including exposed mine wastes, capping materials and surrounding soils.

The sampling design was intended to characterize representative site conditions within each study zone while accounting for the spatial heterogeneity that is typical of mining environments, rather than exhaustively characterizing the spatial variability of the entire mining complexes. The use of replicated composite samples helped reduce the influence of small-scale heterogeneity and provided information on within-site variability. However, these samples cannot be considered independent replicates of restoration treatments at the landscape scale. Consequently, comparisons among sites should be interpreted as site-specific evidence reflecting contrasting restoration conditions rather than as generalizable restoration effects. In addition, no pre-restoration data were available, preventing a direct assessment of temporal changes attributable to restoration interventions.

Zone 1 corresponds to the Brunita (B) mine tailing (~ 8.2 ha, ~ 850,000 m^3^), which contains acidic materials derived from the grinding and metallurgical processing of pyrite, sphalerite, and galena (IGME, 2002; Villar et al., 1991). Partial surface capping was implemented in January 2019 through the application of coarse rock fragments (mine spoils) on the gently sloping tailing surface to reduce erosion and contaminant mobilization. However, the capping was incomplete, and mine waste remained exposed in some areas. Two sampling sites were established on the tailing: B1, located in the upper part of the tailing where capping material was present (capping material), and B2, located in the lower part where mine wastes remained partially exposed (mine waste). A third site was located at the base of the tailing (B3), representing the surrounding soil potentially affected by the dispersion of contaminated materials (Fig. S1, Suppl. Mat.).

Zone 2 corresponds to the Cabezo Rajao (CR) mining complex (~ 280 ha), developed on carbonate-rich lithologies and characterized by the presence of altered mine wastes and surrounding soils. Several tailings within the complex have flat surfaces that were historically capped with calcareous materials and partially afforested (Fig. S1, Suppl. Mat.). Three sampling sites were selected within one tailing and its surroundings: (i) CR1, corresponding to the upper flat surface of the tailing where a carbonate-rich sandy material had been used for capping (capping material); (ii) CR2, located on the tailing slope where the capping layer is incomplete and oxidation-derived secondary minerals were present at the surface (mine waste); and (iii) CR3, located at the base of the tailing in an adjacent agricultural field, with lettuce being cultivated at the time of sampling (surrounding soil) (Fig. S1, Suppl. Mat.).

Zone 3 corresponds to the El Chorrillo (Ch) recreational area (~ 14 ha), afforested with pine trees since the 1980s and located approximately 500 m from a former mining refinery (Lavadero Remunerada) (Fig. S1, Suppl. Mat.). Although this site has also been affected by the historical mining legacy of the district, decades of pine establishment have promoted dense tree cover, litter accumulation on the soil surface, and the development of a comparatively mature soil profile with visible organic matter accumulation. The site was included as an example of a long-established afforested area within the mining district, providing a useful reference context for comparison with nearby mine-affected and restored sites (B1-B3 and CR1-CR3). Soil samples were collected beneath the pine canopy following removal of the surface litter layer.

Zone 4 corresponds to the Las Matildes (M) Mine Interpretation Centre (Fig. S1, Suppl. Mat.), where a demonstration restoration was implemented on a small former tailing (~ 700 m^2^) containing Pb-rich waste. The intervention consisted of sealing the tailing with phyllite rock layers, covering them with clean soil, and afforesting with native shrub species, while a portion of the tailing was intentionally left unrestored to enable comparison between restored and unrestored conditions. Soil samples were collected at three sites: (i) M1, corresponding to the restored area (capping material); (ii) M2, corresponding to the unrestored portion (mine waste); and (iii) M3, located in an agricultural field at the base of the tailing (surrounding soil) (Fig. S1, Suppl. Mat.).

### Sample processing and analyses

Samples were air-dried and passed through a 2-mm sieve prior to analysis. Particle size distribution was determined with a Mastersizer 2000LF laser diffraction analyzer (Malvern Instruments) after dispersion with sodium polyphosphate. Water-holding capacity (WHC) was determined gravimetrically using porous-based glass cylinders following sample saturation for 3 h and free drainage for 2 h (ISO, 1998). Cation exchange capacity (CEC) was determined using 1 M CH_3_COONH_4_ at pH 7 (Chapman, 1965). Briefly, soil samples were saturated with 1 M CH_3_COONH_4_ to replace exchangeable cations with NH_4_^+^. The adsorbed NH_4_^+^ was subsequently displaced with Na^+^ using NaCl, and the released NH_4_^+^ was quantified to estimate CEC. In parallel, ammonium acetate extracts were filtered (0.45 µm nylon membrane filters, WICOM) and analyzed (element–NH_4_^+^) for metal(loid)s (Fe, Mn, Zn, Pb, As, and Cd) and rare earth elements (REEs; Y, La, Ce, Nd, and Gd) by inductively coupled plasma mass spectrometry (ICP-MS; Agilent 7900; detection limit 0.002 mg L^−1^). These concentrations were used to estimate the fraction of elements extractable under ammonium acetate conditions, which is commonly interpreted as an operationally defined exchangeable or weakly bound fraction related to their potential mobility. Aliquots of the sieved samples were finely ground in an agate mortar for the determination of total CaCO_3_, total organic carbon (TOC), and total nitrogen (TN) using an elemental analyzer (CHN 628, LECO Corporation) (ISO, 1995), and for the determination of pseudo-total (element-T) metal(loid)s (Fe, Mn, Zn, Pb, As, and Cd) and REEs (Y, La, Ce, Nd, and Gd). Total metal(loid) and REE concentrations were determined by ICP-MS following acid digestion with HNO_3_ (69%) and HCl (37%) (3:1, v:v) in closed vessels using a microwave digestion system (Milestone UltraWAVE SRC) according to USEPA method 3051A (USEPA, 2007). Following digestion, samples were filtered (Albet 145 ashless filter paper, 7–11 µm). Analytical quality control for metal(loid)s was ensured using the certified reference soil Trace Metals-Sandy Loam 6 CRM043 (Supelco), with recoveries ranging between 80 and 110%.

Mineralogical composition was determined in the ground samples by semi-quantitative analysis of the crystalline fraction using powder X-ray diffraction (XRD) with a Bruker AXS D8-Advance diffractometer equipped with CuKα radiation (40 kV, 30 mA).

Soil:water suspensions (1:2.5, w:v) were prepared using sieved samples and shaken for 2 h. After filtration (0.45 µm, WICOM), pH and electrical conductivity (EC) were measured using a Crison Basic 20 pH meter and Crison Basic 30 conductivity meter, respectively. Water-soluble major ions (Cl^−^, SO_4_^2−^, Na^+^, K^+^, Ca^2+^, and Mg^2+^) were determined by ion chromatography (Metrohm 861, Metrohm AG). Water-extractable (element-W) metal(loid)s (Fe, Mn, Zn, Pb, As, and Cd) and REEs (Y, La, Ce, Nd, and Gd), considered here to represent the fraction readily mobilizable under aqueous conditions, were measured by ICP-MS. Water-soluble organic carbon (WSOC) and water-soluble nitrogen (WSN) were determined using an automatic analyzer (Multi N/C 3100, Analytik Jena). Mineral nitrogen forms were extracted using 1 M KCl (soil:solution 1:5, w:v) by shaking for 1 h, followed by filtration through 0.45 µm nylon membrane syringe filters. Nitrate (NO_3_^−^) concentrations were determined spectrophotometrically at 220 nm, applying a correction for dissolved organic matter measured at 275 nm (APHA et al., 2023). Ammonium (NH_4_^+^) was quantified colorimetrically at 670 nm using the salicylate method described by Nelson (1983). Water-soluble organic nitrogen (WSON) was calculated as WSN minus inorganic N (N–NO_3_^−^ and N–NH_4_^+^). Available phosphorus was extracted with 0.5 M NaHCO_3_ and measured at 825 nm using the ascorbic acid method (Olsen et al., 1954). Absorbance measurements were performed using a PerkinElmer Lambda 25 UV/Vis spectrophotometer.

Soil microbial functionality was assessed through C and N mineralization assays following OECD standardized guidelines. For C mineralization (OECD, 2000a), sieved samples were adjusted to 50% of their WHC and incubated for 28 d at 20 °C in darkness. After incubation, samples were amended with glucose, sealed with a silicone septum, and CO_2_ production was measured every 2 h over a 12-h period using a PBI Dansensor Checkmate II analyzer. For N mineralization (OECD, 2000b), sieved samples were pre-incubated at 50% WHC for 2 d at 20 °C in darkness and amended with lucerne-grass-green-meal (~ 99% organic N). After 0, 7, 14, 21, and 28 d of incubation, subsamples were extracted with 1 M KCl (1:5, 2 h shaking) and analyzed for NO_3_^−^ and NH_4_^+^ as previously indicated. Sample moisture was checked twice weekly throughout the incubation period.

### Statistical analysis

Statistical analyses were performed using IBM SPSS Statistics 24 (SPSS, 2016). Differences were considered statistically significant at *p* ≤ 0.05. Data were transformed when necessary to satisfy the assumptions of normality (Shapiro–Wilk test) and homogeneity of variances (Levene’s test). Differences among groups were assessed using one-way ANOVA followed by Tukey’s HSD post hoc test, except for data from N mineralization assays, which were analyzed using repeated-measures ANOVA followed by Tukey’s HSD test. Pearson’s correlations were calculated to evaluate the relationships among all the assessed parameters.

## Results

### Granulometry, mineralogy and general chemical soil characteristics

Soils across the study zones were predominantly sandy to sandy loam in texture, with sand contents generally ranging from 47 to 87% and silt from 12 to 48%, while clay contents were consistently low (< 9%) (Table S1, Suppl. Mat.). In the Las Matildes zone, the mine waste (M2) and the surrounding soil (M3) showed finer textures, characterized by higher silt proportions (around 60%) and lower sand contents (33–37%).

Mineralogical composition was dominated by primary silicate phases, mainly muscovite (16–72%) and quartz (12–48%), across all zones (Table S1, Suppl. Mat.). Chlorite-serpentine occurred at low to moderate proportions (3–9%) in several sites. In the Brunita zone, only minor mineralogical differences were observed among sites, and secondary sulfate and Fe-(oxyhydr)oxide phases (e.g., epsomite, gypsum, plumbojarosite and goethite) occurred at low proportions. In the Cabezo Rajao zone, mineralogical differences among sites were more pronounced. The mine waste (CR2) exhibited abundant secondary minerals, notably epsomite (14%), kaolinite (12%) and gypsum (4%), whereas the capping material (CR1) and the surrounding soil (CR3) contained carbonate minerals (11–12% calcite and 4–5% dolomite). The soil from El Chorrillo (Ch) contained only trace amounts of secondary phases. In the Las Matildes zone, the mine waste (M2) was characterized by abundant plumbojarosite (15%), dolomite (9%), gypsum (8%) and goethite (5%), while the capping material (M1) and the surrounding soil (M3) showed higher proportions of carbonate minerals (12–13% calcite).

**Fig. 1:**
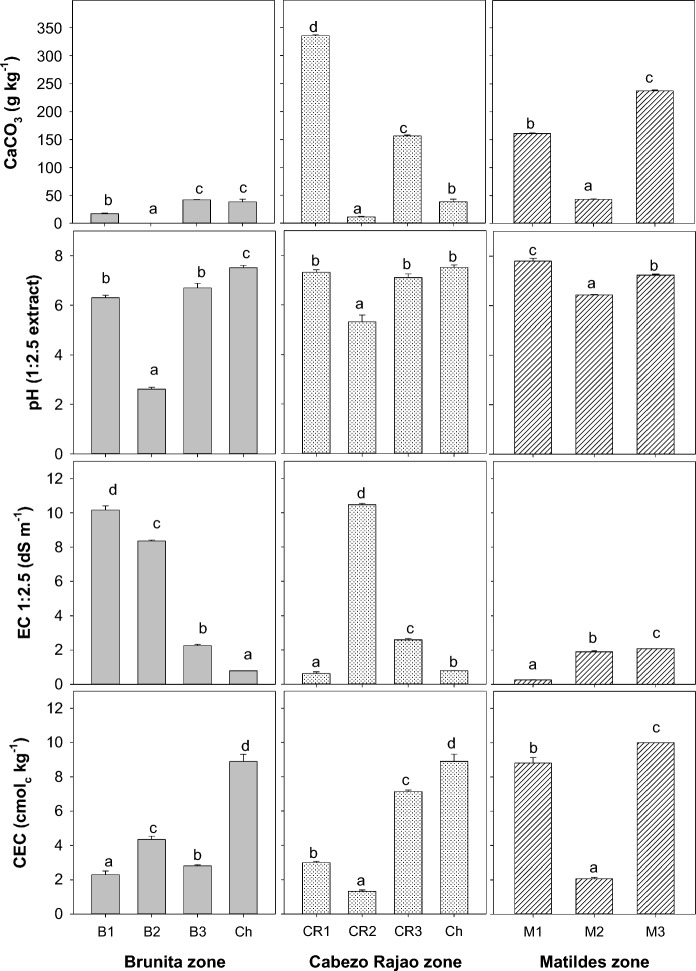
CaCO_3_, pH, electrical conductivity (EC) and cation exchange capacity (CEC). Values are mean ± standard error (n = 5). Different letters indicate significant differences among sampling sites within each study zone (one-way ANOVA followed by Tukey’s HSD post hoc test, *p* ≤ 0.05)

Carbonate contents (CaCO_3_) were generally low at most sites (< 45 g kg^−1^), except in the capping materials and surrounding soils of the Cabezo Rajao (CR1 and CR3) and Las Matildes (M1 and M3), where values exceeded 150 g kg^−1^ (Fig. 1). Soil pH varied widely across the study zones, ranging from strongly acidic to slightly alkaline (Fig. 1). The lowest value was recorded in the Brunita mine waste (B2, pH 2.6), whereas moderately acidic conditions were observed in the Cabezo Rajao mine waste (CR2, pH 5.3), the Brunita capping material (B1, pH 6.3), and the Las Matildes mine waste (M2, pH 6.4). The remaining sites showed near-neutral to slightly alkaline pH values (6.7–7.8). Electrical conductivity (EC) indicated elevated salinity at most sites (2–10.5 dS m^−1^), except for the El Chorrillo soil (Ch) and the capping materials of the Cabezo Rajao (CR1) and Las Matildes (M1) (0.3–0.8 dS m^−1^) (Fig. 1). The most saline sites (B1, B2, and CR2) were characterized by higher SO_4_^2−^ and Mg^2+^ concentrations, whereas Cl^−^, Na^+^, K^+^ and Ca^2+^ predominated in less saline samples (Fig. S2, Suppl. Mat.). Cation exchange capacity (CEC) ranged from extremely low values in the Brunita (B1, B2 and B3) and Cabezo Rajao (CR1 and CR2) sites (≤ 4 cmol_c_ kg^−1^) to low-to-moderate values in the El Chorrillo soil (Ch), the Las Matildes capping material (M1), and the surrounding soils (CR3 and M3) (7–10 cmol_c_ kg^−1^) (Fig. 1).

### Total and extractable metal(loid) concentrations

**Fig. 2:**
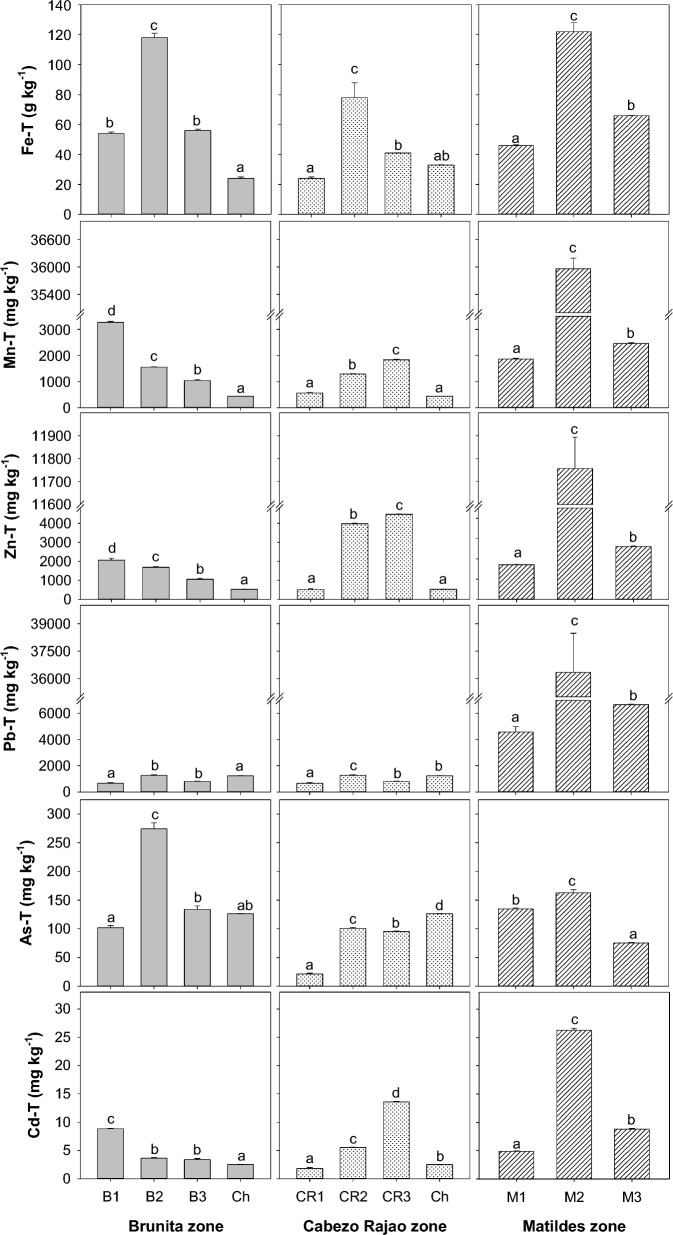
Total contents of Fe, Mn, Zn, Pb, As and Cd. Values are mean ± standard error (n = 5). Different letters indicate significant differences among sampling sites within each study zone (one-way ANOVA followed by Tukey’s HSD post hoc test, *p* ≤ 0.05)

Total metal(loid) concentrations were elevated across all study zones, with maximum values systematically recorded in mine wastes (Fig. 2). The Las Matildes mine waste (M2) showed the highest concentrations overall, reaching 35,960 mg kg^−1^ Mn-T, 11,760 mg kg^−1^ Zn-T, 36,340 mg kg^−1^ Pb-T, and 26 mg kg^−1^ Cd-T, whereas the Brunita mine waste (B2) contained the highest As-T concentration (274 mg kg^−1^). Fe-T concentrations were elevated at all sites (> 24 g kg^−1^), with maxima exceeding 110 g kg^−1^ in B2 and M2 (Fig. 2).

Spatial patterns differed among zones (Fig. 2). In the Brunita zone, Mn-T and Zn-T were higher in the tailing sites (B1 and B2) than in the surrounding soil (B3), while Cd-T peaked in the capping material (B1). In the Cabezo Rajao and Las Matildes zones, total metal(loid) concentrations were consistently higher in the mine wastes (CR2 and M2) and surrounding soils (CR3 and M3) than in the capping materials (CR1 and M1). The soil from El Chorrillo (Ch) showed lower total concentrations overall, although As-T (126 mg kg^−1^) and Pb-T (1238 mg kg^−1^) remained relatively elevated (Fig. 2).

Water- and NH_4_^+^-extractable metal(loid) concentrations varied markedly among study zones and site types (Figs. S3 and S4, Suppl. Mat.), and their distribution among sites differed from that observed for total concentrations. The Brunita zone displayed the highest water-extractable concentrations, particularly in the capping material (B1) and the mine waste (B2) (e.g., up to 1174 mg kg^−1^ Mn–W, 760 mg kg^−1^ Zn–W, and 2.5 mg kg^−1^ Cd–W), together with substantial exchangeable pools (e.g., Mn–NH_4_^+^ up to 129 mg kg^−1^). In the Cabezo Rajao zone, maximum exchangeable concentrations were observed in the mine waste (CR2) and the surrounding soil (CR3), particularly for Zn–NH_4_^+^ (up to 24 mg kg^−1^). In the Las Matildes zone, the mine waste (M2) exhibited the highest concentrations of Pb–W (1.5 mg kg^−1^), Pb–NH_4_^+^ (434 mg kg^−1^), and Cd–NH_4_^+^ (0.5 mg kg^−1^), whereas the surrounding soil (M3) showed the highest As–W (3.6 mg kg^−1^). Extractable concentrations in the El Chorrillo soil (Ch) were generally low, although measurable Pb–NH_4_^+^ (4.5 mg kg^−1^) was detected.

### Total and extractable rare earth element concentrations

**Fig. 3:**
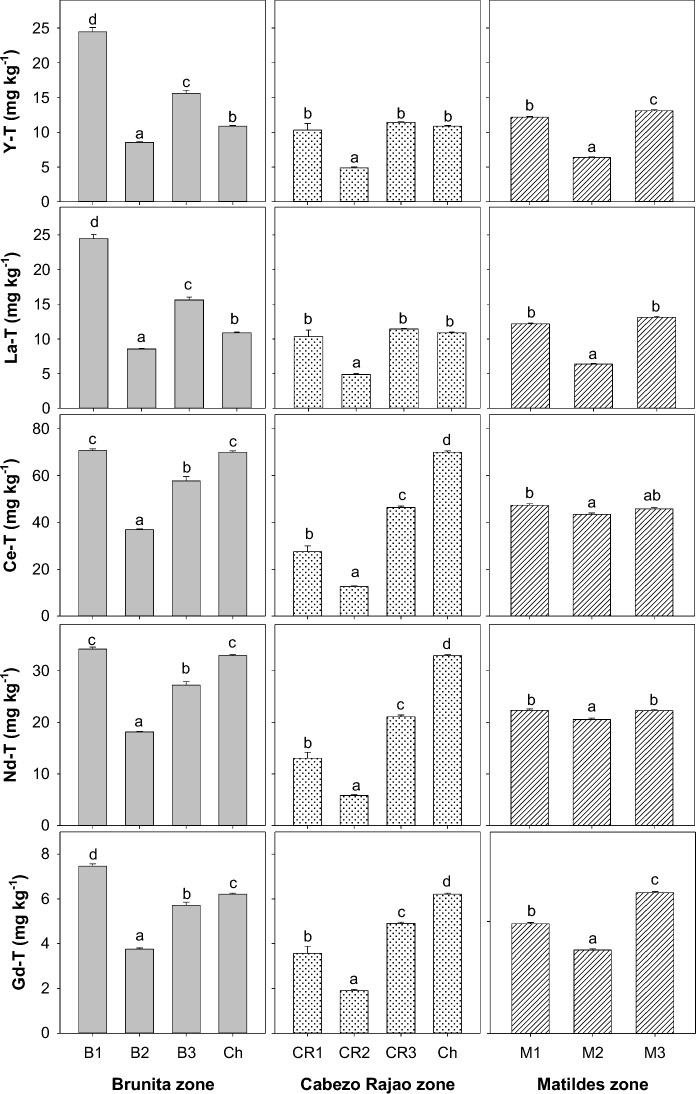
Total contents of Y, La, Ce, Nd and Gd. Values are mean ± standard error (n = 5). Different letters indicate significant differences among sampling sites within each study zone (one-way ANOVA followed by Tukey’s HSD post hoc test, *p* ≤ 0.05)

Total rare earth element (REE) concentrations tended to be lower in the mine wastes (B2, CR2 and M2) than in the capping materials (B1, CR1 and M1) and the surrounding soils (B3, CR3 and M3) (Fig. 3). Variability among zones and among sites within individual zones was generally limited. The highest total concentrations were recorded in the Brunita capping material (B1; e.g., 33 mg kg^−1^ La-T, 71 mg kg^−1^ Ce-T), whereas the lowest values occurred in the Cabezo Rajao mine waste (CR2).

Water-extractable concentrations (Fig. S5, Suppl. Mat.) were substantially higher in the Brunita zone, particularly in the mine waste (B2), where all elements reached maximum values (e.g., Ce–W up to 3086 µg kg^−1^ and Y–W up to 1276 µg kg^−1^), with the next highest values recorded in the capping material (B1). Concentrations in the surrounding soil (B3) and the soil from El Chorrillo (Ch) were substantially lower than those observed in B1 and B2. In the Cabezo Rajao zone, the mine waste (CR2) also exhibited higher water-extractable concentrations than the remaining sites (e.g., 27 µg kg^−1^ Y–W and La–W and 43 µg kg^−1^ Ce-W), although values were one to two orders of magnitude lower than those observed in Brunita. In the Las Matildes zone, overall REE mobility remained limited, with only moderate enrichment observed in the mine waste (M2) (e.g., 28 µg kg^−1^ La–W).

NH_4_^+^-extractable REEs showed a different distribution pattern (Fig. S6, Suppl. Mat.). In the Brunita zone, the highest exchangeable concentrations were recorded in the capping material (B1; e.g., 278 µg kg^−1^ La–NH_4_^+^, 512 µg kg^−1^ Ce-NH_4_^+^, 255 µg kg^−1^ Nd–NH_4_^+^), followed by the mine waste (B2), whereas values in the surrounding soil (B3) and the El Chorrillo soil (Ch) were substantially lower than those observed in B1 and B2. In the Las Matildes zone, the mine waste (M2) exhibited enrichment in La–NH_4_^+^, Ce-NH_4_^+^ and Nd–NH_4_^+^ (405, 112 and 106 µg kg^−1^, respectively), while concentrations in the capping material (M1) and the surrounding soil (M3) were negligible. In the Cabezo Rajao zone, the mine waste (CR2) showed the highest NH_4_^+^-extractable concentrations within the zone, although values remained well below those recorded in Brunita and Las Matildes.

### Soil nutritional and functional parameters

**Fig. 4:**
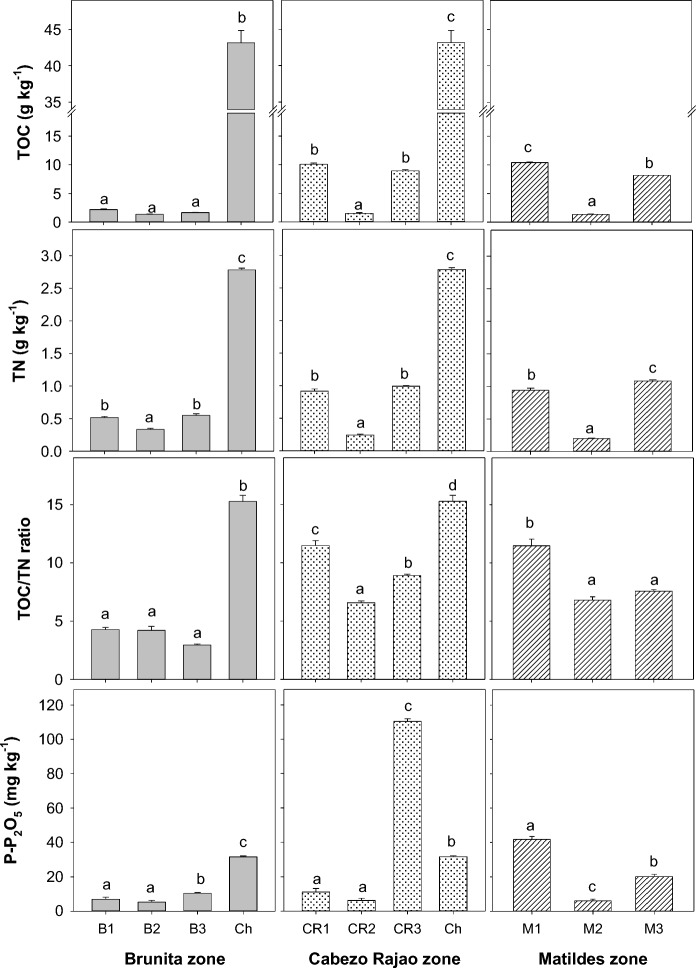
Total organic carbon (TOC), total nitrogen (TN), TOC/TN ratio, and P–P_2_O_5_. Values are mean ± standard error (n = 5). Different letters indicate significant differences among sampling sites within each study zone (one-way ANOVA followed by Tukey’s HSD post hoc test, *p* ≤ 0.05)

Soil carbon and nutrient pools showed marked differences among study zones and site types (Fig. 4). Very low concentrations of TOC (≤ 2 g kg^−1^) and TN (≤ 0.5 g kg^−1^), together with low TOC:TN ratios (3–7), were observed in all sites within the Brunita zone (B1, B2 and B3) and in the mine waste sites in Cabezo Rajao (CR2) and Las Matildes (M2). By contrast, moderate contents of TOC (8–10 g kg^−1^) and TN (≈1 g kg^−1^) contents, with TOC:TN ratios between 8 and 11, were recorded in the capping materials and the surrounding soils of Cabezo Rajao (CR1 and CR3) and Las Matildes (M1 and M3). The soil from El Chorrillo (Ch) exhibited the highest TOC and TN contents (43 g kg^−1^ and 3 g kg^−1^, respectively) and the highest TOC:TN ratio (15). Available phosphorus (P–P_2_O_5_) concentrations were generally low across most sites (< 11 mg kg^−1^), except in the surrounding soils from Cabezo Rajao (CR3, 110 mg kg^−1^) and Las Matildes (M3, 20 mg kg^−1^), the El Chorrillo soil (Ch, 31 mg kg^−1^), and the Las Matildes capping material (M1, 41 mg kg^−1^).

**Fig. 5:**
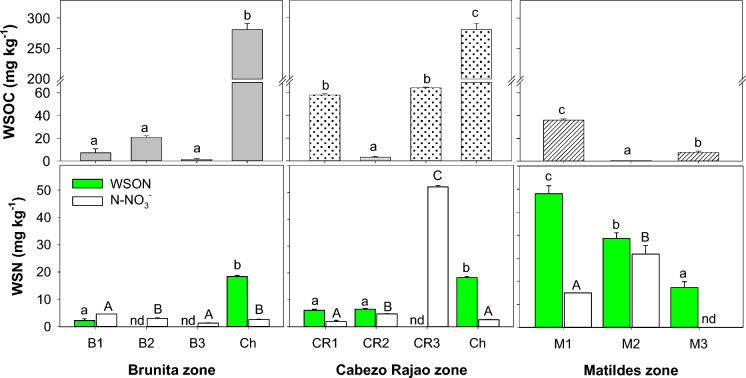
Water soluble organic carbon (WSOC). Water soluble nitrogen (WSN): water soluble organic N (WSON); water soluble inorganic N (N–NO_3_^−^, since N–NH_4_^+^ concentrations were negligible). Values are mean ± standard error (n = 5). Different lower-case letters indicate significant differences among sampling sites within each study zone for WSOC and WSON, and upper-case letters for N–NO_3_^−^ (one-way ANOVA followed by Tukey’s HSD post hoc test, *p* ≤ 0.05). nd (not detected)

Water-soluble organic carbon (WSOC) exhibited considerable spatial variability (Fig. 5). The lowest concentrations were recorded in the Brunita surrounding soil (B3) and the mine wastes from the Cabezo Rajao (CR2) and Las Matildes mine waste (M2) (≤ 3 mg kg^−1^), whereas the highest value occurred in the El Chorrillo soil (Ch, 281 mg kg^−1^). Intermediate WSOC concentrations (20–64 mg kg^−1^) were observed at the remaining sites. Organic forms dominated water-soluble nitrogen (WSN) in most soils (WSON 2–18 mg kg^−1^ in CR1, CR2, Ch, M1, M2 and M3), whereas in B1, B2, B3 and CR3 nitrate represented the dominant soluble N form, with particularly high concentrations in the Cabezo Rajao surrounding soil (CR3, up to 52 mg kg^−1^) (Fig. 5).

**Fig. 6:**
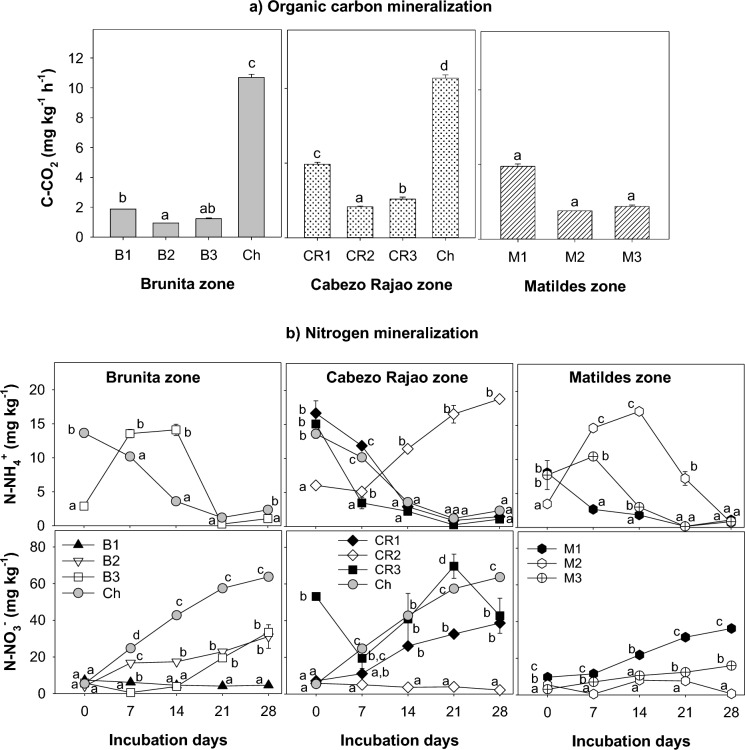
**a** C–CO_2_ emissions during organic carbon mineralization assays. Values are mean ± standard error (n = 5). Different letters indicate significant differences among sampling sites within each study zone (one-way ANOVA followed by Tukey’s HSD post-hoc test, *p* ≤ 0.05). **b** N–NO_3_^−^ and N–NH_4_^+^ dynamics during nitrogen mineralization assays. Values are mean ± standard error (n = 5). For each incubation day, different letters indicate differences among sampling sites within each study zone (repeated-measures ANOVA followed by Tukey’s HSD post-hoc test, *p* ≤ 0.05)

Carbon mineralization rates (C–CO_2_ emissions) remained low (< 2.6 mg kg^−1^ h^−1^) in all sites within the Brunita zone (B1, B2 and B3) and in the mine wastes and surrounding soils of Cabezo Rajao (CR2 and CR3) and Las Matildes (M2 and M3) (Fig. 6). Higher carbon mineralization rates were recorded in the capping materials of Cabezo Rajao (CR1) and Las Matildes (M1) (4.9 mg kg^−1^ h^−1^), while the highest values were recorded in the soil from El Chorrillo (Ch, 11 mg kg^−1^ h^−1^).

Nitrogen mineralization patterns differed clearly among zones and site types (Fig. 6). In the Brunita zone, N–NH_4_^+^ was only detected in the surrounding soil (B3) between days 7 and 14 (up to 13 mg kg^−1^), whereas N–NO_3_^−^ concentrations increased after day 14 (from 19 to 33 mg kg^−1^). In the capping material (B1) and the mine waste (B2), N–NH_4_^+^ was not detected during incubation, while N–NO_3_^−^ remained low in B1 (4–7 mg kg^−1^) but increased progressively in B2 (from 4 to 31 mg kg^−1^). In the El Chorrillo soil (Ch), N–NH_4_^+^ decreased over time (from 13 to 2 mg kg^−1^) and N–NO_3_^−^ increased markedly (from 5 to 64 mg kg^−1^). Similar trends were observed in the Cabezo Rajao capping material (CR1) and the surrounding soil (CR3). In contrast, in the mine waste (CR2), N–NH_4_^+^ increased after day 7 (from 5 to 18 mg kg^−1^), whereas N–NO_3_^−^ remained low throughout the incubation (2–6 mg kg^−1^). In the Las Matildes zone, the capping material (M1) and the surrounding soil (M3) showed decreasing N-NH_4_^+^ (from 8 to 1 mg kg^−1^) and increasing N–NO_3_^−^ (from 3–9 to 15–36 mg kg^−1^) concentrations, while in the mine waste (M2), N–NH_4_^+^ increased transiently between days 7 and 14 (up to 14–17 mg kg^−1^) and N–NO_3_^−^ concentrations remained low (6–8 mg kg^−1^).

## Discussion

### Mineralogical and geochemical factors influencing metal(loid) distribution and mobility

The mineralogical composition of soils collected from the different sites (capping materials, mine wastes and surrounding soils) within each study zone (Table S1, Suppl. Mat.) reflects the combined influence of native geological materials, mining residues and restoration inputs. Primary minerals such as quartz and phyllosilicates (muscovite and chlorite-serpentine), common in the regional lithology (Martínez-López et al., 2021), predominated in almost all samples regardless of zone or site. In contrast, iron sulfides were only identified in mine waste from the Las Matildes zone (M2), mainly as pyrrhotite, indicating the partial preservation of primary sulfides at this site, whereas their absence elsewhere suggests that extensive alteration has occurred.

Calcite and dolomite were mainly detected in the capping materials and surrounding soils from the Cabezo Rajao and Las Matildes zones (CR1 and CR3; M1 and M3). This mineral assemblage is consistent with the abundance of carbonate lithologies in the area and helps explain the high CaCO_3_ contents and neutral to slightly alkaline pH values observed at these sites (Fig. 1), thereby providing substantial buffering capacity against acidification. In contrast, clear enrichment in secondary sulfate and Fe-(oxyhydr)oxide phases was observed in the mine wastes from the Cabezo Rajao and Las Matildes zones (CR2 and M2), including minerals such as epsomite, gypsum, plumbojarosite, goethite and hematite. These phases result from supergenic alteration of sulfide- and Fe-rich materials exposed to atmospheric conditions (Burke et al., 2023; Robles-Arenas & Candela, 2010; Rodríguez-Pacheco et al., 2023). Sulfide oxidation is the primary process driving acid mine drainage formation, generating acidity and sulfate ions (Rodríguez-Pacheco et al., 2023). The resulting secondary phases differ considerably in both their solubility and their role in metal(loid) retention. While hydrated sulfate minerals such as epsomite may represent more labile phases, poorly soluble Pb-bearing minerals such as plumbojarosite, together with Fe-(oxyhydr)oxides such as goethite and hematite, can act as important sinks for metal(loid)s released during sulfide weathering (Navarro et al., 2008), thereby limiting Pb mobility despite high total concentrations. In addition, the occurrence of gypsum reflects the reaction between sulfate and Ca^2+^ ions, which may locally attenuate acidity (Burke et al., 2023). In the Brunita zone, by contrast, mineralogical differences among sites were relatively minor and secondary phases were present only at low proportions. This suggests that the extremely high metal(loid) solubility observed in this zone is primarily driven by unfavorable physicochemical conditions (very low pH and high salinity) rather than by mineralogical contrasts alone (Fig. 1 and Fig. S3, Suppl. Mat.).

Total metal(loid) concentrations measured in this study largely exceeded the recently established regional Generic Values of Reference (GVRs) for soils of the Murcia Region (BORM, 2025), which define threshold values for the protection of human health. The comparison was based on the GVRs established for the category “other land uses” (i.e., land uses other than industrial and urban). These thresholds (e.g., 600 mg kg^−1^ for Mn, 705 for Zn, 115 for Pb, 4 for Cd, and 13 for As) were exceeded at most sites, indicating widespread high to extremely high contamination levels. Total Fe contents were also extremely high, particularly in the mine wastes, and showed significant positive correlations with Mn-T, Zn-T, Cd-T, Pb-T and As-T (*p* < 0.001) (Table S2, Suppl. Mat.). García-Lorenzo et al. (2012) distinguished between primary contamination associated with mine tailings, secondary contamination affecting surrounding areas through wind and water erosion, and tertiary contamination derived from ongoing alteration and mobilization processes. The widespread exceedance of GVR thresholds in both mine wastes and surrounding soils indicates that these different contamination processes coexist in the study zones, reflecting the long-term persistence and spatial extent of mining impacts. These findings agree with previous studies reporting extensive dispersion of metal(loid)s throughout the La Unión-Sierra de Cartagena mining district (Conesa & Schulin, 2010).

Among the studied zones, Las Matildes was the most severely affected by Mn, Zn, Pb and Cd (Fig. 2). Maximum concentrations were found in the mine waste (M2), although the capping material from the restored area (M1) and the adjacent agricultural field (M3) also showed elevated values. The elevated metal contents in M1 may be attributed to: (i) the use of a capping material that was not free of contamination, possibly sourced from impacted nearby areas; (ii) continuous input of contaminated particles from the unrestored portion of the tailing (M2), or (iii) a combination of both processes. The extremely high total metal concentrations together with the absence of vegetation cover in M2 indicate that the exposed mine waste continues to act as an active source of metal dispersion. Particularly noteworthy are the elevated total metal(loid) concentrations observed in soil from the El Chorrillo recreational area, especially for As, which raises concerns regarding potential risks for both the environment and human health.

Beyond total concentration, the assessment of available metal(loid) fractions provides a more informative basis for evaluating contaminant mobility, potential environmental risks and the effectiveness of restoration measures. Water-soluble and exchangeable fractions are commonly preferred indicators of metal(loid) availability (McLaughlin et al., 2000). Water extraction primarily targets the readily soluble fraction, which tends to increase under acidic conditions, whereas neutral salt solutions (e.g., CH_3_COONH_4_) target the exchangeable pool. The amounts extracted by both procedures are strongly influenced by soil properties such as pH, mineralogy and texture (Giller et al., 2009; Palumbo-Roe et al., 2009; Zúñiga-Vázquez et al., 2023). While total metal(loid) concentrations provide an indication of contamination severity, they do not necessarily reflect potential bioavailability or ecological risk. In this context, the water- and NH_4_^+^-extractable fractions assessed in this study can be considered operational proxies for mobile and potentially bioavailable pools. These fractions are particularly useful for evaluating potential exposure pathways, including plant uptake, leaching to surface and groundwater, and the transport of soluble metal(loid) forms. Therefore, the combined assessment of total and extractable concentrations, together with key soil properties controlling metal(loid) mobility, provides a more realistic basis for evaluating potential contaminant transfer across environmental compartments and ecological risks at the study sites. In this study, total concentrations of Mn and Zn were not correlated with their corresponding water- and NH_4_^+^-extractable fractions, whereas significant relationships were observed between Cd-T and Cd–NH_4_^+^ and between Pb-T and both Pb-NH_4_^+^ and Pb–W (*p* < 0.001; Table S2, Suppl. Mat.), highlighting that total contents alone are often poor predictors of metal(loid) availability. However, Fe-T was significantly correlated with several available fractions (Zn–W, Cd–W, Pb–W, Cd–NH_4_^+^ and Pb–NH_4_^+^; *p* ≤ 0.001; Table S2, Suppl. Mat.), underscoring the central role of Fe phases in controlling metal(loid) retention and mobility.

Expressing extractable concentrations as percentages of total content provides additional insight into the factors controlling metal(loid) mobility. For the NH_4_^+^-extractable fraction, Zn, As and Pb generally represented less than 0.5% of their total concentrations, whereas Cd showed higher proportions, reaching up to 5.8% in the Brunita mine waste (B2). Similarly, Mn exhibited higher exchangeable fractions in the Brunita sites (3–4%) than in the remaining soils (< 0.5%). These results indicate that, despite elevated total concentrations, only small proportions of most elements were present in the exchangeable pool, with Cd showing comparatively greater mobility.

Water extraction yielded higher concentrations of metal(loid)s than ammonium acetate extraction in acidic sites (B1, B2, CR2 and M2), whereas this pattern was not observed in soils with neutral to basic pH (Fig. 1 and Figs. S3 and S4, Suppl. Mat.). This interpretation was further supported by expressing water-extractable concentrations as percentages of total contents. Particularly high soluble fractions were observed for Cd (up to 67%), Zn (up to 45%) and Mn (up to 40%) in the Brunita sites and, to a lesser extent, in CR2. In contrast, Pb represented less than 0.01% of total concentrations in all soils, while As remained below 0.1% except in M3 (4.8%). These findings demonstrate that the highest soluble fractions were not necessarily associated with the highest total concentrations and reinforce the importance of soil properties, particularly pH, salinity and buffering capacity, as primary controls of metal(loid) mobility. Soil pH showed significant negative correlations with Mn–W, Zn–W and Cd–W (*p* ≤ 0.001), whereas no such relationship was observed for the NH_4_^+^-extractable fractions (Table S2, Suppl. Mat.). This behavior reflects the strong pH dependence of the water-extractable fraction, whereas the neutral pH of the ammonium acetate extractant buffers this effect and reduces the influence of soil acidity on extractable metal concentrations. Previous leaching experiments have demonstrated that both pH and CaCO_3_ content exert strong control over the solubility of Mn, Zn and Cd, with higher pH and carbonate contents leading to lower solubility (Simón et al., 2010). Accordingly, significant negative correlations were found between the water-extractable concentrations of these metals and both pH and CaCO_3_ (*p* ≤ 0.02; Table S2, Suppl. Mat.).

In contrast to Mn, Zn and Cd, Pb and As exhibited different geochemical behavior. The environmental relevance of these patterns should be considered in relation to the absolute concentrations of their water-soluble and exchangeable fractions, which were generally low for some elements and sites (Figs. S3 and S4, Suppl. Mat.).

Total Pb concentrations were significantly correlated with both Pb–W and Pb–NH_4_^+^ (*p* < 0.001), while these available fractions were independent of pH and CaCO_3_ but strongly correlated with Fe-T (*p* < 0.001) (Table S2, Suppl. Mat.). This is consistent with previous studies indicating that Fe-(oxyhydr)oxides play a more relevant role than carbonates in controlling Pb dissolution and mobilization (Palumbo-Roe et al., 2009; Simón et al., 2010). Accordingly, in sites showing high extractable Pb concentrations (e.g., the Las Matildes zone), these geochemical controls may have considerable environmental significance, as reflected by the combination of high total, water-extractable and exchangeable concentrations (Fig. 2 and Figs. S3 and S4, Suppl. Mat.). In this zone, Pb is associated with plumbojarosite (PbFe_6_(SO_4_)_4_(OH)_12_), which reached up to 15% in the mine waste (Table S1, Suppl. Mat.). Plumbojarosite is a secondary mineral commonly formed in oxidized Pb-rich systems and has also been reported at high concentrations in saline efflorescences of the area (Alcolea-Rubio et al., 2022). These efflorescences enhance metal dispersion due to their high solubility and susceptibility to wind and water transport. The significant positive correlations observed between SO_4_^2−^ concentrations and most total, water- and NH_4_^+^-extractable metal(loid)s (*p* ≤ 0.048; Table S2, Suppl. Mat.) support the role of soluble salts in metal(loid) dispersion.

Total As concentrations were positively correlated with Fe-T (*p* < 0.001), but not with total contents of other metals. In contrast to other metals, As–NH_4_^+^ was negatively correlated with Fe-T and positively correlated with pH (*p* ≤ 0.001), while As–W was positively correlated with CaCO_3_ (*p* < 0.05) (Table S2, Suppl. Mat.). This behavior is consistent with previous findings showing that CaCO_3_ promotes the predominance of HAsO_4_^2−^ and enhances As mobility, whereas Fe-(oxyhydr)oxides effectively reduce its availability in carbonate-poor systems (Simón et al., 2010, 2014). Given the relatively low extractable As concentrations found in most sites, these relationships likely have limited environmental significance.

### Rare earth elements: occurrence and mobility

Rare earth elements (REEs) have received increasing attention due to their extensive industrial applications and growing environmental relevance. Unlike classical metal(loid)s associated with sulfide mining, elevated REE concentrations in soils may occur independently of mining contamination and often reflect a predominantly geogenic origin (Pagano et al., 2015). Importantly, total REE concentrations alone are poor indicators of environmental risk, as their environmental relevance is largely controlled by extractable fractions and by soil chemical properties (Brioschi et al., 2013; Kulaksız & Bau, 2013; Li et al., 2018).

Total concentrations of Y, La, Ce, Nd and Gd across the study zones fall within the ranges commonly reported for the Earth’s crust and for a wide variety of soils worldwide (Hu et al., 2006; Ramos et al., 2016; Tao et al., 2022; Tyler, 2004). The values observed in this study fall within the background ranges reported for Spanish soils: 5.3–128 mg kg^−1^ (La), 11–246 mg kg^−1^ (Ce), 4.6–132 mg kg^−1^ (Nd), and 0.9–24 mg kg^−1^ (Gd) (Ramos et al., 2016). Comparable ranges have also been reported in mining-affected areas with similar lithological settings, such as the nearby Cerro de San Cristóbal district in the Mazarrón mining area (Murcia Region, SE Spain), located approximately 60 km southwest of the Cartagena-La Unión district and developed under comparable geological conditions (Azizi et al., 2022, 2023). Collectively, these findings indicate that historical mining activity in the La Unión-Sierra de Cartagena district has not resulted in significant REE enrichment in mine wastes or surrounding soils, despite strong contamination by other metal(loid)s. This interpretation is further supported by the significant positive correlations among total REE concentrations (*p* < 0.001) and their lack of correlation with most of the metal(loid)s analyzed (Table S3, Suppl. Mat.), consistent with a predominantly geogenic origin.

In agreement with their predominantly geogenic origin, REEs generally showed very low extractability and limited mobility across the study sites. Ammonium acetate-extractable concentrations always represented less than 1.9% of total concentrations, while water-extractable fractions were generally below 0.5%, except for Y (14% in B2), La (3–6% in B1 and B2), Ce (1.5–8% in B1 and B2) and Nd (2.5% in B2). REE availability across the study sites was strongly controlled by key soil properties, in agreement with established geochemical behavior (Brioschi et al., 2013; Ramos et al., 2016; Wells & Wells, 2001). Increasing pH reduces REE solubility through the formation of hydroxides and carbonates (Cao et al., 2001; González et al., 2014), while organic matter, aluminosilicates, and iron and manganese oxides can provide effective retention surfaces (Beckwith & Butler, 1983; Pang et al., 2002). Consistent with these mechanisms, most water-extractable REE concentrations showed significant negative correlations with pH, clay content and CaCO_3_ content and positive correlations with sand content (Table S4, Suppl. Mat.), indicating a higher mobilization potential in coarse-textured, weakly buffered soils. In addition, both water- and NH_4_^+^-extractable REE concentrations were positively correlated with SO_4_^2−^ ions (Table S4, Suppl. Mat.), suggesting an association with soluble salts and efflorescences, as also observed for several metal(loid)s.

These patterns help explain the marked increases in extractable REE concentrations observed particularly in the Brunita zone, and, to a lesser extent, in the mine wastes from Cabezo Rajao (CR2) and Las Matildes (M2), where very low pH, high salinity and coarse texture favor mobilization processes. These results demonstrate that, even in the absence of anomalously high total concentrations, REE mobility can be locally enhanced under severely degraded soil conditions. Consequently, REE behavior in these systems appears to be governed primarily by soil properties rather than by mining-derived enrichment. The inclusion of REEs in integrated geochemical assessments may therefore be particularly relevant under extreme edaphic conditions, where mobilization processes are intensified.

### Soil nutritional and functional status: implications for restoration effectiveness

Soil functionality indicators, including nutrient availability, microbial activity and organic matter mineralization, are commonly used to assess restoration effectiveness because they integrate the physical, chemical and biological processes that sustain ecosystem functioning (Anderson & Domsch, 2000; Bastida et al., 2008). In this study, these indicators revealed a clear functional gradient across the study sites, ranging from the more functionally developed soil at El Chorrillo (Ch) to mining-affected sites exhibiting varying degrees of functional impairment and recovery. In particular, restoration measures in the Cabezo Rajao and Las Matildes zones (CR1 and M1) involved the addition of relatively clean, fine materials combined with leveling and the establishment of vegetation cover, whereas the Brunita intervention (B1) was limited to a thin surface layer of coarse mine-derived fragments aimed primarily at reducing erosion, without organic amendments or materials capable of supporting soil development.

Soil organic matter and nutrient-related indicators clearly reflected these differences. Extremely low TOC and TN contents, together with limited WSOC and WSON, were observed in the mine wastes (B2, CR2 and M2) and in several capped or surrounding soils (particularly in B1 and B3) (Fig. 4 and Fig. 5), indicating depleted organic matter pools and restricted substrate availability for microbial metabolism. TOC/TN ratios were also below values typically reported for Mediterranean soils (Lagomarsino et al., 2009), consistent with poorly developed organic matter pools. By contrast, the El Chorrillo soil (Ch) exhibited substantially higher TOC, TN, WSOC and WSON contents, reflecting a more developed and functionally active soil system associated with long-term vegetation cover and continuous organic inputs (Getino-Álvarez et al., 2023), both of which support microbial activity under semi-arid conditions (Maestre & Cortina, 2004). These values are consistent with those previously reported for well-developed pine forest soils surrounding mine tailings within the same mining district (Álvarez-Rogel et al., 2021), supporting the interpretation of Ch as a long-established and functionally active afforested soil. However, As and Pb concentrations indicate that Ch still reflects the influence of the historical mining legacy of the area. Furthermore, differences between Ch and the other mining-affected sites are likely influenced not only by contamination and restoration status, but also by the presence of long-established forest vegetation and associated soil development processes. Consequently, Ch should be regarded as a relatively developed forest soil within the mining district when interpreting comparisons with other sites. Intermediate values recorded in the capped and surrounding soils from the Cabezo Rajao and Las Matildes zones (CR1, CR3, M1 and M3) indicate partial recovery of organic-matter-related properties, whereas the Brunita zone showed consistently poor conditions across all site types.

Available phosphorus followed a similar pattern, with very low concentrations in most mine wastes and in the Brunita zone (Fig. 4), likely due to sorption onto Fe-(oxyhydr)oxides, which reduces P bioavailability (Simón et al., 2010). Higher values in the surrounding agricultural soil from Cabezo Rajao (CR3) probably reflect fertilization inputs rather than intrinsic functional recovery.

Biological indicators provided further support for this gradient of functional recovery. Carbon mineralization rates were substantially lower in the Brunita zone and in several mine wastes and surrounding soils (Fig. 6), reflecting the combined effects of low organic matter availability, unfavorable physicochemical conditions and, in some cases, high availability of potentially toxic metal(loid)s (Giller et al., 2009). Elevated salinity may also have constrained microbial activity in some sites through osmotic stress effects. In contrast, the soil from El Chorrillo (Ch) exhibited the highest carbon mineralization rates, while the capped soils from the Cabezo Rajao and Las Matildes zones (CR1 and M1) showed intermediate values, confirming partial but incomplete biological recovery.

Nitrogen mineralization dynamics followed a similar pattern: the El Chorrillo soil (Ch), the capped soils from Cabezo Rajao and Las Matildes zones (CR1 and M1) and their surrounding soils (CR3 and M3) showed evidence of active nitrification, whereas the mine wastes (B2, CR2 and M2) displayed limited or irregular N transformations, indicative of impaired microbial functioning. The capping material from the Brunita zone (B1) behaved similarly to the exposed mine wastes, highlighting the persistence of substantial constraints on microbial activity at this site. This contrasts with the response observed in the capped soils from Cabezo Rajao (CR1) and Las Matildes (M1), where more favorable pH conditions, higher organic matter contents and established vegetation supported relatively active N cycling. This pattern is consistent with the characteristics of the intervention applied at Brunita, which focused primarily on physical stabilization rather than soil development, without incorporating organic amendments or materials capable of improving buffering capacity and supporting revegetation. These conditions may have limited the recovery of microbial processes at this site.

## Conclusions

This study shows that legacy contamination remains severe across the La Unión-Sierra de Cartagena mining district, including in areas where restoration actions were previously implemented. Total concentrations of Mn, Zn, Cd, Pb and As greatly exceeded the regional GVRs not only in mine wastes but also in surrounding soils and, in some cases, in capping materials. These findings indicate that contamination pressures extend beyond the tailings themselves and that the impacts of historical mining persist at the landscape scale despite previous restoration efforts.

The assessment of extractable metal(loid) fractions showed that total concentrations alone were poor predictors of contaminant mobility, which was primarily controlled by soil properties, particularly pH, buffering capacity and salinity. Rare earth elements showed no evidence of mining-related enrichment, although enhanced mobility was observed locally under extreme edaphic conditions.

Soil functional indicators revealed clear differences in soil recovery among sites representing contrasting restoration conditions. While some capped soils showed evidence of partial recovery of organic matter pools, nutrient availability and microbial activity, sites where restoration actions were largely restricted to surface stabilization remained functionally constrained and comparable to exposed mine wastes. These findings suggest that physical stabilization alone may be insufficient to achieve substantial soil recovery in semi-arid mining environments and highlight the importance of restoration approaches that promote both contaminant stabilization and soil development. Carbonate-rich materials may represent a suitable option to provide buffering capacity and reduce the mobility of several cationic metals by preventing acidification. However, in systems affected by As or other oxyanion-forming elements, additional sorptive materials may be necessary to further limit contaminant mobility.

## Supplementary Information

Below is the link to the electronic supplementary material.

Supplementary file1 (DOCX 2732 KB)

## Data Availability

No datasets were generated or analysed during the current study.
