# Supplementary material for: Soil chemical and functional indicators reveal limitations of restoration measures in abandoned metal mine soils of SE Spain: implications for ongoing and future management
Source: Environ Geochem Health. 2026 Jul 22;48(11):477. doi: 10.1007/s10653-026-03378-3 (PMC13391719; doi:10.1007/s10653-026-03378-3)
Supplement: Supplementary file 1 — Supplementary file1 (DOCX 2732 KB) [file 10653_2026_3378_MOESM1_ESM.docx]

SUPPLEMENTARY MATERIAL

**Soil chemical and functional indicators reveal limitations of restoration measures in abandoned metal mine soils of SE Spain: implications for ongoing and future remediation**

Matías Ceacero-Moreno, José Álvarez-Rogel and M. Nazaret González-Alcaraz*

Department of Agricultural Engineering of the E.T.S.I.A., Technical University of Cartagena member of European University of Technology EUT+, 30203 Cartagena, Spain.

*Corresponding author: M.N. González Alcaraz. Department of Agricultural Engineering of the E.T.S.I.A., Technical University of Cartagena member of European University of Technology EUT+, 30203 Cartagena, Spain. Phone: +34. 968.325.411; Email: nazaret.gonzalez@upct.es

Supplementary material summary: 1 cover page, 6 figures, and 4 tables.


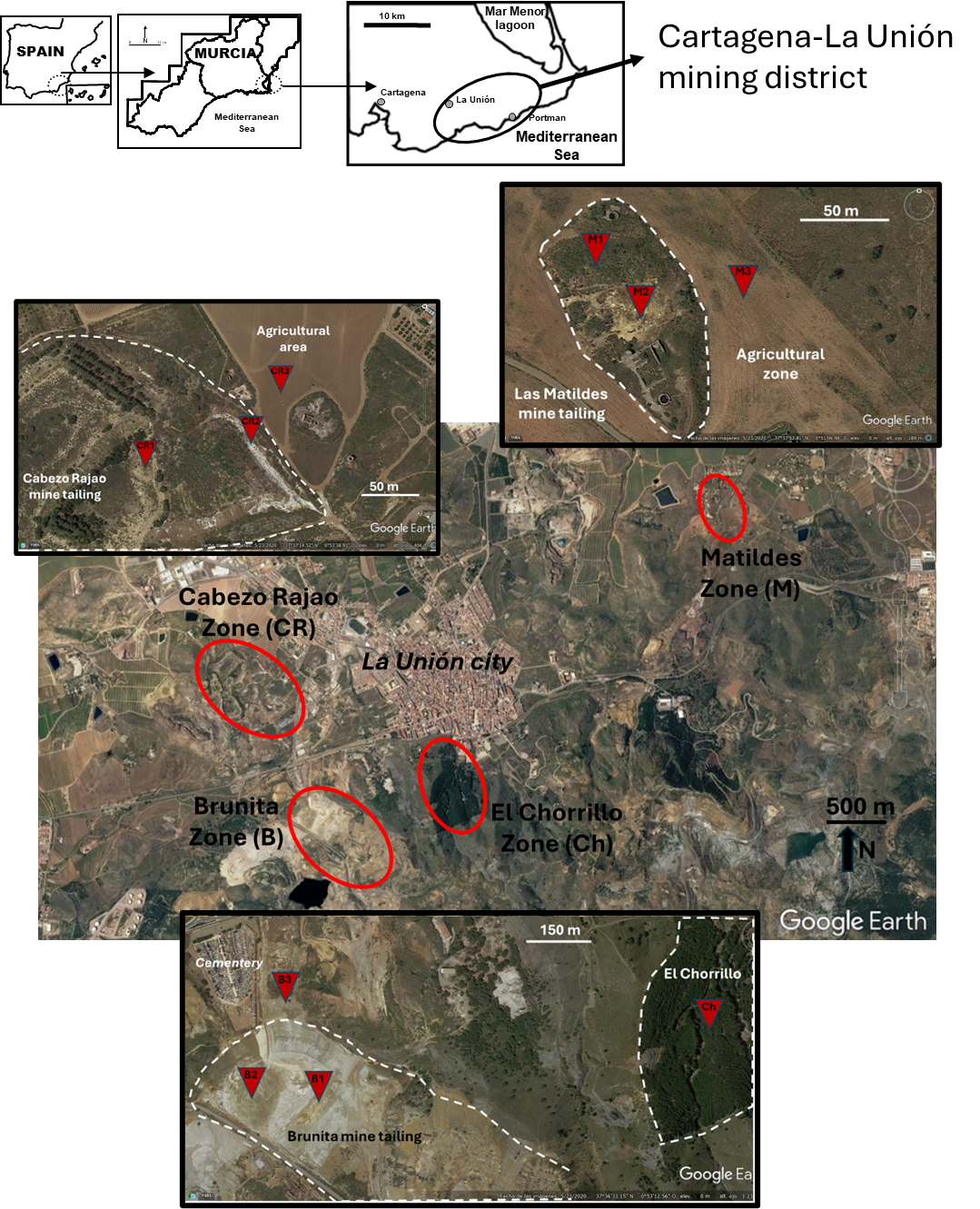


**Figure S1**. Location map of the studied zones and sampling sites.

**Figure S2**. Major ions of the 1:2.5 soil:water extracts. Values are mean ± standard error (n = 5). CR: Cabezo Rajao zone; B: Brunita zone; Ch: El Chorrillo zone; M: Las Matildes zone. Different letters indicate significant differences among sampling sites within each study zone (one-way ANOVA followed by Tukey’s HSD post hoc test, p ≤ 0.05).

**Figure S3**. Water-extractable Mn, Zn, Pb, As and Cd (1:2.5 soil:water extractions). Values are mean ± standard error (n = 5). CR: Cabezo Rajao zone; B: Brunita zone; Ch: El Chorrillo zone; M: Las Matildes zone. Different letters indicate significant differences among sampling sites within each study zone (one-way ANOVA followed by Tukey’s HSD post hoc test, p ≤ 0.05).

**Figure S4**. Mn, Zn, Pb, As and Cd extracted with 1 M CH_3_COONH_4_. Values are mean ± standard error (n = 5). CR: Cabezo Rajao zone; B: Brunita zone; Ch: El Chorrillo zone; M: Las Matildes zone. Different letters indicate significant differences among sampling sites within each study zone (one-way ANOVA followed by Tukey’s HSD post hoc test, p ≤ 0.05).

**Figure S5**. Water-extractable Y, La, Ce, Nd and Gd (1:2.5 soil:water extractions). Values are mean ± standard error (n = 5). CR: Cabezo Rajao zone; B: Brunita zone; Ch: El Chorrillo zone; M: Las Matildes zone. Different letters indicate significant differences among sampling sites within each study zone (one-way ANOVA followed by Tukey’s HSD post hoc test, p ≤ 0.05).

**Figure S6**. Y, La, Ce, Nd and Gd extracted with 1 M CH_3_COONH_4_. Values are mean ± standard error (n = 5). CR: Cabezo Rajao zone; B: Brunita zone; Ch: El Chorrillo zone; M: Las Matildes zone. Different letters indicate significant differences among sampling sites within each study zone (one-way ANOVA followed by Tukey’s HSD post hoc test, p ≤ 0.05).**Table S1**. Granulometry (percentage of sand, silt, and clay) and mineralogy (percentage of the main minerals) of the samples analyzed. Values are mean (n = 5). CR: Cabezo Rajao zone; B: Brunita zone; Ch: El Chorrillo zone; M: Las Matildes zone.

|  | **B1** | **B2** | **B3** | **CR1** | **CR2** | **CR3** | **Ch** | **M1** | **M2** | **M3** |
| --- | --- | --- | --- | --- | --- | --- | --- | --- | --- | --- |
| **Granulometry (%)** |  |  |  |  |  |  |  |  |  |  |
| Sand | 63 | 87 | 84 | 52 | 67 | 47 | 59 | 70 | 33 | 37 |
| Silt | 35 | 12 | 15 | 44 | 28 | 48 | 38 | 23 | 59 | 57 |
| Clay | 2 | 1 | 1 | 4 | 5 | 5 | 3 | 7 | 8 | 6 |
| **Mineralogy (%)** |  |  |  |  |  |  |  |  |  |  |
| Muscovite | 72 | 65 | 69 | 53 | 16 | 21 | 50 | 34 | 17 | 18 |
| Quartz | 12 | 19 | 20 | 22 | 45 | 48 | 38 | 36 | 30 | 46 |
| Chlorite-serpentine | 7 | 9 | 7 | 6 |  | 4 | 3 | 4 |  | 5 |
| Epsomite | 5 | 2 |  |  | 14 | 3 |  |  |  | 3 |
| Calcite |  |  | 2 | 11 |  | 12 | 3 | 12 |  | 13 |
| Dolomite |  |  |  | 4 |  | 5 |  | 1 | 9 | 0.7 |
| Plumbojarosite | 0.7 | 1 | 0.2 |  | 3 |  |  | 0.8 | 15 | 3 |
| Gypsum | 1 | 0.8 | 0.5 |  | 4 | 0.4 |  | 0.1 | 8 | 2 |
| Kaolinite |  |  |  |  | 12 | 3 | 3 | 1 | 4 |  |
| Clintonite | 2 | 1 | 2 | 3 | 2 | 1 | 1 | 4 |  | 3 |
| Goethite | 0.3 | 2 | 0.4 | 1 | 3 | 0.9 |  | 0.8 | 5 | 1 |
| Hematite |  |  |  |  |  |  |  | 0.6 | 3 |  |
| Clinochlore |  |  |  |  | 2 |  |  |  |  | 1 |
| Albite |  |  |  |  |  | 1 | 1 |  | 3 | 4 |
| Montmorillonite |  |  |  |  |  | 0.8 | 0.6 | 1 |  | 0.8 |
| Talc |  |  |  |  |  |  | 0.5 |  |  |  |
| Edenite |  |  |  |  |  |  |  | 2 | 4 |  |
| Pyrrhotite |  |  |  |  |  |  |  |  | 2 |  |
| Aluminite |  |  |  |  |  |  |  | 3 |  |  |

**Table S2.** Pearson correlations (two tails) among CaCO_3_, pH, total (T), water extractable (W) and ammonium acetate extractable (NH4) metals and As, and water-soluble sulphate (SO4). ** Significant at p ≤ 0.01; * Significant at p ≤ 0.05. N (number of observations).

|  |  | CaCO3 | pH | Mn-T | Zn-T | As-T | Cd-T | Pb-T | Mn-W | Zn-W | As-W | Cd-W | Pb-W | Mn-NH4 | Zn-NH4 | As-NH4 | Cd-NH4 | Pb-NH4 | SO4 | Fe-T |
| --- | --- | --- | --- | --- | --- | --- | --- | --- | --- | --- | --- | --- | --- | --- | --- | --- | --- | --- | --- | --- |
| CaCO3 | Corr. | 1 | .548^**^ | -.209 | -.240 | -.687^**^ | -.168 | -.153 | **-.432^*^** | **-.423^*^** | **.423^*^** | **-.609^**^** | .235 | -.435^*^ | -.256 | .260 | -.457^*^ | -.206 | -.629^**^ | -.532** |
|  | Sig. |  | .002 | .277 | .210 | .000 | .384 | .428 | **.019** | **.020** | **.031** | **.001** | .270 | .021 | .189 | .190 | .014 | .292 | .003 | .003 |
|  | N | 30 | 30 | 29 | 29 | 29 | 30 | 30 | 29 | 30 | 26 | 27 | 24 | 28 | 28 | 27 | 28 | 28 | 20 | 29 |
| pH | Corr. |  | 1 | .000 | -.050 | -.722^**^ | .063 | .038 | **-.455^**^** | **-.835^**^** | .156 | **-.905^**^** | .157 | -.223 | -.207 | **.503^**^** | -.135 | .040 | -.652^**^ | -.703** |
|  | Sig. |  |  | 1.000 | .733 | .000 | .672 | .797 | **.001** | **.000** | .335 | **.000** | .328 | .173 | .206 | **.001** | .412 | .809 | .000 | .000 |
|  | N |  |  | 48 | 48 | 48 | 48 | 48 | 49 | 50 | 40 | 42 | 41 | 39 | 39 | 38 | 39 | 39 | 35 | 48 |
| Mn-T | Corr. |  |  | 1 | .928^**^ | .209 | .899^**^ | .978^**^ | -.112 | -.029 | -.042 | -.049 | .847^**^ | -.100 | -.090 | -.285 | .853^**^ | .995^**^ | .600^**^ | .606** |
|  | Sig. |  |  |  | .000 | .155 | .000 | .000 | .455 | .844 | .800 | .762 | .000 | .543 | .585 | .083 | .000 | .000 | .000 | .000 |
|  | N |  |  |  | 48 | 48 | 48 | 48 | 47 | 48 | 38 | 40 | 39 | 39 | 39 | 38 | 39 | 39 | 35 | 48 |
| Zn-T | Corr. |  |  |  | 1 | .165 | .959^**^ | .917^**^ | -.153 | .005 | .005 | -.074 | .776^**^ | -.157 | .239 | -.206 | .876^**^ | .918^**^ | .452^**^ | .622** |
|  | Sig. |  |  |  |  | .261 | .000 | .000 | .305 | .975 | .978 | .652 | .000 | .341 | .143 | .215 | .000 | .000 | .006 | .000 |
|  | N |  |  |  |  | 48 | 48 | 48 | 47 | 48 | 38 | 40 | 39 | 39 | 39 | 38 | 39 | 39 | 35 | 48 |
| As-T | Corr. |  |  |  |  | 1 | .106 | .201 | .253 | .676^**^ | -.170 | .683^**^ | .058 | .082 | -.137 | -.276 | .210 | .188 | .337^*^ | **.729**** |
|  | Sig. |  |  |  |  |  | .474 | .172 | .086 | .000 | .309 | .000 | .726 | .621 | .404 | .094 | .200 | .252 | .048 | **.000** |
|  | N |  |  |  |  |  | 48 | 48 | 47 | 48 | 38 | 40 | 39 | 39 | 39 | 38 | 39 | 39 | 35 | 48 |
| Cd-T | Corr. |  |  |  |  |  | 1 | .874^**^ | -.049 | -.078 | .075 | -.143 | .762^**^ | -.026 | .109 | -.128 | **.889^**^** | .872^**^ | .177 | .511** |
|  | Sig. |  |  |  |  |  |  | .000 | .743 | .598 | .655 | .380 | .000 | .873 | .510 | .445 | **.000** | .000 | .310 | .000 |
|  | N |  |  |  |  |  | 48 | 48 | 47 | 48 | 38 | 40 | 39 | 39 | 39 | 38 | 39 | 39 | 35 | 48 |
| Pb-T | Corr. |  |  |  |  |  |  | 1 | -.209 | -.052 | .082 | -.104 | **.906^**^** | -.202 | -.060 | -.247 | .802^**^ | **.985^**^** | .566^**^ | .615 |
|  | Sig. |  |  |  |  |  |  |  | .159 | .726 | .626 | .521 | **.000** | .218 | .719 | .135 | .000 | **.000** | .000 | .000 |
|  | N |  |  |  |  |  |  |  | 47 | 48 | 38 | 40 | 39 | 39 | 39 | 38 | 39 | 39 | 35 | 48 |
| Mn-W | Corr. |  |  |  |  |  |  |  | 1 | .523^**^ | -.198 | .742^**^ | -.344^*^ | .898^**^ | -.153 | -.438^**^ | .194 | -.189 | **.624^**^** | .172 |
|  | Sig. |  |  |  |  |  |  |  |  | .000 | .227 | .000 | .030 | .000 | .360 | .007 | .244 | .256 | **.000** | .249 |
|  | N |  |  |  |  |  |  |  |  | 49 | 39 | 41 | 40 | 39 | 38 | 37 | 38 | 38 | **34** | 47 |
| Zn-W | Corr. |  |  |  |  |  |  |  |  | 1 | -.047 | .827^**^ | -.156 | .231 | .095 | -.444^**^ | .055 | -.076 | **.628^**^** | .620 |
|  | Sig. |  |  |  |  |  |  |  |  |  | .772 | .000 | .331 | .156 | .566 | .005 | .737 | .648 | **.000** | .000 |
|  | N |  |  |  |  |  |  |  |  |  | 40 | 42 | 41 | 39 | 39 | 38 | 39 | 39 | **35** | 48 |
| As-W | Corr. |  |  |  |  |  |  |  |  |  | 1 | -.295 | .416^*^ | -.159 | .059 | -.199 | -.100 | -.081 | .341 | .085 |
|  | Sig. |  |  |  |  |  |  |  |  |  |  | .096 | .014 | .371 | .742 | .267 | .574 | .650 | .076 | .612 |
|  | N |  |  |  |  |  |  |  |  |  |  | 33 | 34 | 34 | 34 | 33 | 34 | 34 | 28 | 38 |
| Cd-W | Corr. |  |  |  |  |  |  |  |  |  |  | 1 | -.277 | .509^**^ | -.003 | -.534^**^ | .134 | -.103 | **.745^**^** | .587 |
|  | Sig. |  |  |  |  |  |  |  |  |  |  |  | .097 | .002 | .987 | .001 | .449 | .563 | **.000** | .000 |
|  | N |  |  |  |  |  |  |  |  |  |  |  | 37 | 34 | 34 | 33 | 34 | 34 | **30** | 40 |
| Pb-W | Corr. |  |  |  |  |  |  |  |  |  |  |  | 1 | -.313 | -.163 | -.240 | .630^**^ | .839^**^ | -.110 | **.519** |
|  | Sig. |  |  |  |  |  |  |  |  |  |  |  |  | .076 | .363 | .186 | .000 | .000 | .594 | **.000** |
|  | N |  |  |  |  |  |  |  |  |  |  |  |  | 33 | 33 | 32 | 33 | 33 | 26 | **39** |
| Mn-NH4 | Corr. |  |  |  |  |  |  |  |  |  |  |  |  | 1 | -.112 | -.376^*^ | .283 | -.157 | .539^**^ | .038 |
|  | Sig. |  |  |  |  |  |  |  |  |  |  |  |  |  | .496 | .020 | .081 | .340 | .003 | .818 |
|  | N |  |  |  |  |  |  |  |  |  |  |  |  |  | 39 | 38 | 39 | 39 | 28 | 39 |
| Zn-NH4 | Corr. |  |  |  |  |  |  |  |  |  |  |  |  |  | 1 | -.031 | .159 | -.095 | **.466^*^** | .133 |
|  | Sig. |  |  |  |  |  |  |  |  |  |  |  |  |  |  | .855 | .333 | .566 | **.012** | .421 |
|  | N |  |  |  |  |  |  |  |  |  |  |  |  |  |  | 38 | 39 | 39 | **28** | 39 |
| As-NH4 | Corr. |  |  |  |  |  |  |  |  |  |  |  |  |  |  | 1 | -.351^*^ | -.255 | **-.650^**^** | **-.583** |
|  | Sig. |  |  |  |  |  |  |  |  |  |  |  |  |  |  |  | .031 | .122 | **.000** | **.000** |
|  | N |  |  |  |  |  |  |  |  |  |  |  |  |  |  |  | 38 | 38 | **28** | **38** |
| Cd-NH4 | Corr. |  |  |  |  |  |  |  |  |  |  |  |  |  |  |  | 1 | .826^**^ | **.599^**^** | .599 |
|  | Sig. |  |  |  |  |  |  |  |  |  |  |  |  |  |  |  |  | .000 | **.001** | .000 |
|  | N |  |  |  |  |  |  |  |  |  |  |  |  |  |  |  |  | 39 | **28** | 39 |
| Pb-NH4 | Corr. |  |  |  |  |  |  |  |  |  |  |  |  |  |  |  |  | 1 | -.285 | **.579** |
|  | Sig. |  |  |  |  |  |  |  |  |  |  |  |  |  |  |  |  |  | .141 | **.000** |
|  | N |  |  |  |  |  |  |  |  |  |  |  |  |  |  |  |  |  | 28 | **39** |
| SO4 | Corr. |  |  |  |  |  |  |  |  |  |  |  |  |  |  |  |  |  | 1 | .677 |
|  | Sig. |  |  |  |  |  |  |  |  |  |  |  |  |  |  |  |  |  |  | .000 |
|  | N |  |  |  |  |  |  |  |  |  |  |  |  |  |  |  |  |  |  | 35 |

**Table S3.** Pearson correlations (two tails) among total contest of Mn, Zn, As, Cd, Pb, Y, La, Ce, Nd and Gd. ** Significant at p ≤ 0.01; * Significant at p ≤ 0.05. Number of observations (48).

|  |  | Mn-T | Zn-T | As-T | Cd-T | Pb-T | Y-T | La-T | Ce-T | Nd-T | Gd-T |
| --- | --- | --- | --- | --- | --- | --- | --- | --- | --- | --- | --- |
| Mn-T | Corr. | 1 | **.928^**^** | .209 | **.899^**^** | **.978^**^** | **-.295^*^** | -.026 | -.026 | -.254 | -.194 |
|  | Sig. |  | **.000** | .155 | **.000** | **.000** | **.042** | .863 | .860 | .081 | .186 |
| Zn-T | Corr. |  | 1 | .165 | **.959^**^** | **.917^**^** | **-.389^**^** | -.198 | -.191 | **-.380^**^** | **-.323^*^** |
|  | Sig. |  |  | .261 | **.000** | **.000** | **.006** | .178 | .195 | **.008** | **.025** |
| As-T | Corr. |  |  | 1 | .106 | .201 | -.193 | .102 | .087 | -.001 | -.092 |
|  | Sig. |  |  |  | .474 | .172 | .188 | .489 | .557 | .996 | .532 |
| Cd-T | Corr. |  |  |  | 1 | **.874^**^** | -.171 | .011 | .019 | -.151 | -.082 |
|  | Sig. |  |  |  |  | **.000** | .246 | .939 | .896 | .304 | .582 |
| Pb-T | Corr. |  |  |  |  | 1 | **-.365^*^** | -.075 | -.075 | **-.294^*^** | -.229 |
|  | Sig. |  |  |  |  |  | **.011** | .614 | .612 | **.042** | .118 |
| Y-T | Corr. |  |  |  |  |  | 1 | **.742^**^** | **.728^**^** | **.780^**^** | **.863^**^** |
|  | Sig. |  |  |  |  |  |  | **.000** | **.000** | **.000** | **.000** |
| La-T | Corr. |  |  |  |  |  |  | 1 | **.998^**^** | **.961^**^** | **.938^**^** |
|  | Sig. |  |  |  |  |  |  |  | **.000** | **.000** | **.000** |
| Ce-T | Corr. |  |  |  |  |  |  |  | 1 | **.965^**^** | **.932^**^** |
|  | Sig. |  |  |  |  |  |  |  |  | **.000** | **.000** |
| Nd-T | Corr. |  |  |  |  |  |  |  |  | 1 | **.960^**^** |
|  | Sig. |  |  |  |  |  |  |  |  |  | **.000** |
| Gd-T | Corr. |  |  |  |  |  |  |  |  |  | 1 |

**Table S4.** Pearson correlations (two tails) among CaCO_3_, pH, total (T), water extractable (W) and ammonium acetate extractable (NH4) rare earth elements, and water-soluble sulphate (SO4). ** Significant at p ≤ 0.01; * Significant at p ≤ 0.05. N (number of observations).

|  |  | CaCO3 | pH | Y-T | La-T | Ce-T | Nd-T | Gd-T | Y-W | La-W | Ce-W | Nd-W | Gd-W | Y-NH4 | La-NH4 | Ce-NH4 | Nd-NH4 | Gd-NH4 | SO4 |
| --- | --- | --- | --- | --- | --- | --- | --- | --- | --- | --- | --- | --- | --- | --- | --- | --- | --- | --- | --- |
| CaCO3 | Corr. | 1 | **.548^**^** | -.042 | -.235 | -.228 | -.089 | -.003 | -.312 | **-.477^**^** | **-.429^*^** | **-.394^*^** | -.378 | -.456 | -.361 | -.404 | **-.454^*^** | **-.444^*^** | **-.629^**^** |
|  | Sig. |  | **.002** | .828 | .220 | .235 | .645 | .988 | .094 | **.008** | **.018** | **.031** | .069 | .057 | .083 | .062 | **.029** | **.044** | **.003** |
|  | N |  | 30 | 29 | 29 | 29 | 29 | 29 | 30 | 30 | 30 | 30 | 24 | 18 | 24 | 22 | 23 | 21 | 20 |
| pH | Corr. |  | 1 | .241 | **.305^*^** | **.333^*^** | **.359^*^** | **.374^**^** | **-.772^**^** | **-.739^**^** | **-.864^**^** | **-.882^**^** | **-.894^**^** | **-.439^*^** | -.033 | -.168 | -.195 | -.215 | **-.652^**^** |
|  | Sig. |  |  | .099 | **.035** | **.021** | **.012** | **.009** | **.000** | **.000** | **.000** | **.000** | **.000** | **.028** | .849 | .349 | .269 | .244 | **.000** |
|  | N |  |  | 48 | 48 | 48 | 48 | 48 | 50 | 50 | 50 | 47 | 37 | 25 | 36 | 33 | 34 | 31 | 35 |
| Y-T | Corr. |  |  | 1 | **.742^**^** | **.728^**^** | **.780^**^** | **.863^**^** | -.062 | **.367^*^** | .071 | -.051 | -.083 | **.598^**^** | .198 | **.725^**^** | **.639^**^** | **.667^**^** | .014 |
|  | Sig. |  |  |  | **.000** | **.000** | **.000** | **.000** | .674 | **.010** | .631 | .740 | .634 | **.002** | .246 | **.000** | **.000** | **.000** | .937 |
|  | N |  |  |  | 48 | 48 | 48 | 48 | 48 | 48 | 48 | 45 | 35 | 25 | 26 | 33 | 34 | 31 | 35 |
| La-T | Corr. |  |  |  | 1 | **.998^**^** | **.961^**^** | **.938^**^** | -.063 | .210 | .021 | -.054 | -.096 | .352 | .265 | **.488^**^** | **.467^**^** | **.444^*^** | -.282 |
|  | Sig. |  |  |  |  | **.000** | **.000** | **.000** | .670 | .151 | .888 | .724 | .584 | .085 | .118 | **.004** | **.005** | **.012** | .101 |
|  | N |  |  |  |  | 48 | 48 | 48 | 48 | 48 | 48 | 45 | 35 | 25 | 26 | 33 | 34 | 31 | 35 |
| Ce-T | Corr. |  |  |  |  | 1 | **.965^**^** | **.932^**^** | -.087 | .172 | -.014 | -.085 | -.123 | .313 | .248 | **.456^**^** | **.435^*^** | **.407^*^** | -.314 |
|  | Sig. |  |  |  |  |  | **.000** | **.000** | .557 | .243 | .927 | .577 | .481 | .128 | .144 | **.008** | **.010** | **.023** | .067 |
|  | N |  |  |  |  |  | 48 | 48 | 48 | 48 | 48 | 45 | 35 | 25 | 26 | 33 | 34 | 31 | 35 |
| Nd-T | Corr. |  |  |  |  |  | 1 | **.960^**^** | -.071 | .175 | .000 | -.067 | -.082 | .322 | .044 | **.422^*^** | **.361^*^** | **.361^*^** | **-.361^*^** |
|  | Sig. |  |  |  |  |  |  | **.000** | .631 | .234 | .998 | .661 | .640 | .116 | .798 | **.014** | **.036** | **.046** | **.033** |
|  | N |  |  |  |  |  |  | 48 | 48 | 48 | 48 | 45 | 35 | 25 | 26 | 33 | 34 | 31 | 35 |
| Gd-T | Corr. |  |  |  |  |  |  | 1 | -.112 | .191 | -.029 | -.118 | -.155 | **.409^*^** | .178 | **.552^**^** | **.495^**^** | **.492^**^** | -.260 |
|  | Sig. |  |  |  |  |  |  |  | .447 | .193 | .844 | .442 | .375 | **.042** | .299 | **.001** | **.003** | **.005** | .131 |
|  | N |  |  |  |  |  |  |  | 48 | 48 | 48 | 45 | 35 | 25 | 26 | 33 | 34 | 31 | 35 |
| Y-W | Corr. |  |  |  |  |  |  |  | 1 | **.757^**^** | **.874^**^** | **.877^**^** | **.866^**^** | **.403^*^** | -.062 | .123 | .120 | .152 | **.369^*^** |
|  | Sig. |  |  |  |  |  |  |  |  | **.000** | **.000** | **.000** | **.000** | **.046** | .720 | .496 | .498 | .414 | **.029** |
|  | N |  |  |  |  |  |  |  |  | 50 | 50 | 47 | 37 | 25 | 36 | 33 | 34 | 31 | 35 |
| La-W | Corr. |  |  |  |  |  |  |  |  | 1 | **.926^**^** | **.852^**^** | **.822^**^** | **.845^**^** | .211 | **.657^**^** | **.632^**^** | **.689^**^** | **.590^**^** |
|  | Sig. |  |  |  |  |  |  |  |  |  | **.000** | **.000** | **.000** | **.000** | .218 | **.000** | **.000** | **.000** | **.000** |
|  | N |  |  |  |  |  |  |  |  |  | 50 | 47 | 37 | 25 | 36 | 33 | 34 | 31 | 35 |
| Ce-W | Corr. |  |  |  |  |  |  |  |  |  | 1 | **.987^**^** | **.979^**^** | **.639^**^** | .036 | **.358^*^** | **.349^*^** | **.403^*^** | **.480^**^** |
|  | Sig. |  |  |  |  |  |  |  |  |  |  | **.000** | **.000** | **.001** | .836 | **.041** | **.043** | **.024** | **.004** |
|  | N |  |  |  |  |  |  |  |  |  |  | 47 | 37 | 25 | 36 | 33 | 34 | 31 | 35 |
| Nd-W | Corr. |  |  |  |  |  |  |  |  |  |  | 1 | **.999^**^** | **.533^**^** | -.041 | .221 | .219 | .270 | **.395^*^** |
|  | Sig. |  |  |  |  |  |  |  |  |  |  |  | **.000** | **.006** | .816 | .217 | .213 | .141 | **.025** |
|  | N |  |  |  |  |  |  |  |  |  |  |  | 37 | 25 | 36 | 33 | 34 | 31 | 35 |
| Gd-W | Corr. |  |  |  |  |  |  |  |  |  |  |  | 1 | .445 | -.176 | .116 | .113 | .181 | .314 |
|  | Sig. |  |  |  |  |  |  |  |  |  |  |  |  | .064 | .391 | .574 | .574 | .377 | .119 |
|  | N |  |  |  |  |  |  |  |  |  |  |  |  | 25 | 36 | 33 | 34 | 31 | **35** |
| Y-NH4 | Corr. |  |  |  |  |  |  |  |  |  |  |  |  | 1 | **.462^*^** | **.946^**^** | **.920^**^** | **.965^**^** | **.528^*^** |
|  | Sig. |  |  |  |  |  |  |  |  |  |  |  |  |  | **.020** | **.000** | **.000** | **.000** | **.020** |
|  | N |  |  |  |  |  |  |  |  |  |  |  |  |  | 25 | 23 | 24 | 21 | 19 |
| La-NH4 | Corr. |  |  |  |  |  |  |  |  |  |  |  |  |  | 1 | **.618^**^** | **.744^**^** | **.628^**^** | **.499^**^** |
|  | Sig. |  |  |  |  |  |  |  |  |  |  |  |  |  |  | **.000** | **.000** | **.000** | **.006** |
|  | N |  |  |  |  |  |  |  |  |  |  |  |  |  |  | 32 | 33 | 30 | 29 |
| Ce-NH4 | Corr. |  |  |  |  |  |  |  |  |  |  |  |  |  |  | 1 | **.983^**^** | **.987^**^** | **.490^*^** |
|  | Sig. |  |  |  |  |  |  |  |  |  |  |  |  |  |  |  | **.000** | **.000** | **.013** |
|  | N |  |  |  |  |  |  |  |  |  |  |  |  |  |  |  | 33 | 31 | 25 |
| Nd-NH4 | Corr. |  |  |  |  |  |  |  |  |  |  |  |  |  |  |  | 1 | **.987^**^** | **.521^**^** |
|  | Sig. |  |  |  |  |  |  |  |  |  |  |  |  |  |  |  |  | **.000** | **.006** |
|  | N |  |  |  |  |  |  |  |  |  |  |  |  |  |  |  |  | 31 | 26 |
| Gd-NH4 | Corr. |  |  |  |  |  |  |  |  |  |  |  |  |  |  |  |  | 1 | **.512^*^** |
|  | Sig. |  |  |  |  |  |  |  |  |  |  |  |  |  |  |  |  |  | **.010** |
|  | N |  |  |  |  |  |  |  |  |  |  |  |  |  |  |  |  |  | 24 |
| SO4 | Corr. |  |  |  |  |  |  |  |  |  |  |  |  |  |  |  |  |  | 1 |
